# Esophageal Rupture Associated With COVID-19: A Novel Case Report

**DOI:** 10.7759/cureus.12256

**Published:** 2020-12-24

**Authors:** Patrick Meloy, Amit Bhambri

**Affiliations:** 1 Emergency Medicine, Emory University School of Medicine, Atlanta, USA; 2 Emergency Medicine, Swedish Hospital - Part of NorthShore University HealthSystem, Chicago, USA

**Keywords:** mallory-weiss tear, covid-19 pneumonia, covid-19, esophageal rupture

## Abstract

Emergency departments (EDs) are the primary driver for hospital admissions in the United States (US), and that trend is likely to continue through the ongoing severe acute respiratory syndrome coronavirus 2 (SARS-CoV-2) pandemic. As the US continues to experience rampant community spread, coronavirus disease 2019 (COVID-19) will likely present in increasingly variable ways to the EDs. We present a case of Mallory-Weiss tear and esophageal perforation, which was likely caused by COVID-19 pneumonia. This case is notably the first of its kind that we have seen reported in the COVID-19-related literature. Clinicians should be vigilant about the various complications of COVID-19 and continue to exercise caution when seeing and treating these patients.

## Introduction

The number of emergency department (ED) visits reached 139 million in 2017 [1]. Given the ED’s role as the primary driver of hospital admissions, accounting for 70% of hospital admissions in 2018 [2], emergency physicians (EPs) are most likely to be the first healthcare providers for patients infected with severe acute respiratory syndrome coronavirus 2 (SARS-CoV-2), which causes coronavirus disease 2019 (COVID-19). Though the specific nature of the spread of SARS-CoV-2 is still being studied, it is clear that droplets, aerosols, and fomite transmission appear to be the primary drivers of the ongoing pandemic [3]. Due to the respiratory nature of the disease, the most common presentations of COVID-19 appear to be fever and cough, with some patients presenting with chest pain, dyspnea, sore throat, diarrhea, hyposmia, hypogeusia, headache, and multiple other symptoms [4]. Since the Centers for Disease Control and Prevention (CDC) estimates that 2.5% of the United States (US) population has already been infected with COVID-19 [5], EPs should expect to see COVID-19 patients present with a wide variety of complaints. In this case, a 92-year-old female patient presented to the ED complaining of sudden-onset diffuse abdominal pain, nausea, and vomiting. The patient underwent testing in the ED, which was largely unremarkable. Given her symptoms, she was admitted to the hospital for observation. Once admitted, she continued to experience multiple episodes of coughing and vomiting, and she was subsequently found to have an acute Mallory-Weiss tear with full-thickness esophageal perforation, or Boerhaave syndrome. Imaging and additional lab testing also confirmed acute COVID-19 infection. Unfortunately, despite resuscitative attempts, the patient ultimately succumbed to her illness and died during her hospitalization. This case is unique as Boerhaave syndrome is most commonly found as a result of iatrogenic injuries, such as the ones caused by upper endoscopy, nasogastric tube insertion, or other surgical procedures [6,7]. Forceful vomiting can be a presenting symptom in Boerhaave syndrome, and patients are also known to have coughing episodes and hiccups preceding their diagnosis [6]. Esophageal rupture has not been reported in the COVID-19 literature, and we believe this case to be the first reported incidence of Boerhaave syndrome directly associated with symptomatic COVID-19 infection.

## Case presentation

A 92-year-old female with a medical history significant for hypertension, congestive heart failure, aortic stenosis, dementia, and osteoarthritis presented to the ED with sudden-onset nausea, vomiting, and abdominal pain, which had started approximately three hours prior to the presentation. The patient had no previous history of alcohol use or liver disease; she was not a tobacco user and was compliant with her daily aspirin (81 mg), lisinopril (2.5 mg), and furosemide (40 mg) prescriptions. The patient was hypotensive at 97/56 mmHg with a heart rate of 80 bpm. She was afebrile at 37.1 ℃ and was saturating 97% on room air. She appeared acutely ill. Her physical examination revealed epigastric and right upper quadrant abdominal tenderness associated with involuntary guarding.

The patient was seen immediately and was administered a 1-liter normal saline IV bolus, 4 mg of IV ondansetron, and 75 mcg of IV fentanyl. A comprehensive metabolic panel, complete blood count, troponin level, lipase, coagulation panel, and lactic acid were sent, and initial labs were remarkable for creatinine of 1.34 mg/dL, lactic acid of 2.8 mmol/L, normal troponin (<0.03 ng/mL), prothrombin time of 12.9 seconds, international normalized ratio (INR) of 1.1, white blood cell (WBC) count of 3.5 x 10^9^/L, and hemoglobin (Hb) of 9.8 gm/dL. Initial electrocardiogram (EKG) revealed normal sinus rhythm at a rate of 86/min, left axis deviation, no ST-segment changes, and was unchanged when compared to prior EKGs. Due to the severity of her appearance, diminished history secondary to dementia, and the tenderness of her abdomen, an aortic injury was suspected, and the patient underwent a CT angiogram of the chest, abdomen, and pelvis. Imaging did not reveal any obvious cause of her symptoms, and it showed a normal aorta, mildly enlarged heart, a previous cholecystectomy, but no acute pathology. The patient continued to have abdominal tenderness and discomfort. She was given additional doses of fentanyl for symptomatic relief and was admitted to the hospital.

**Figure 1:**
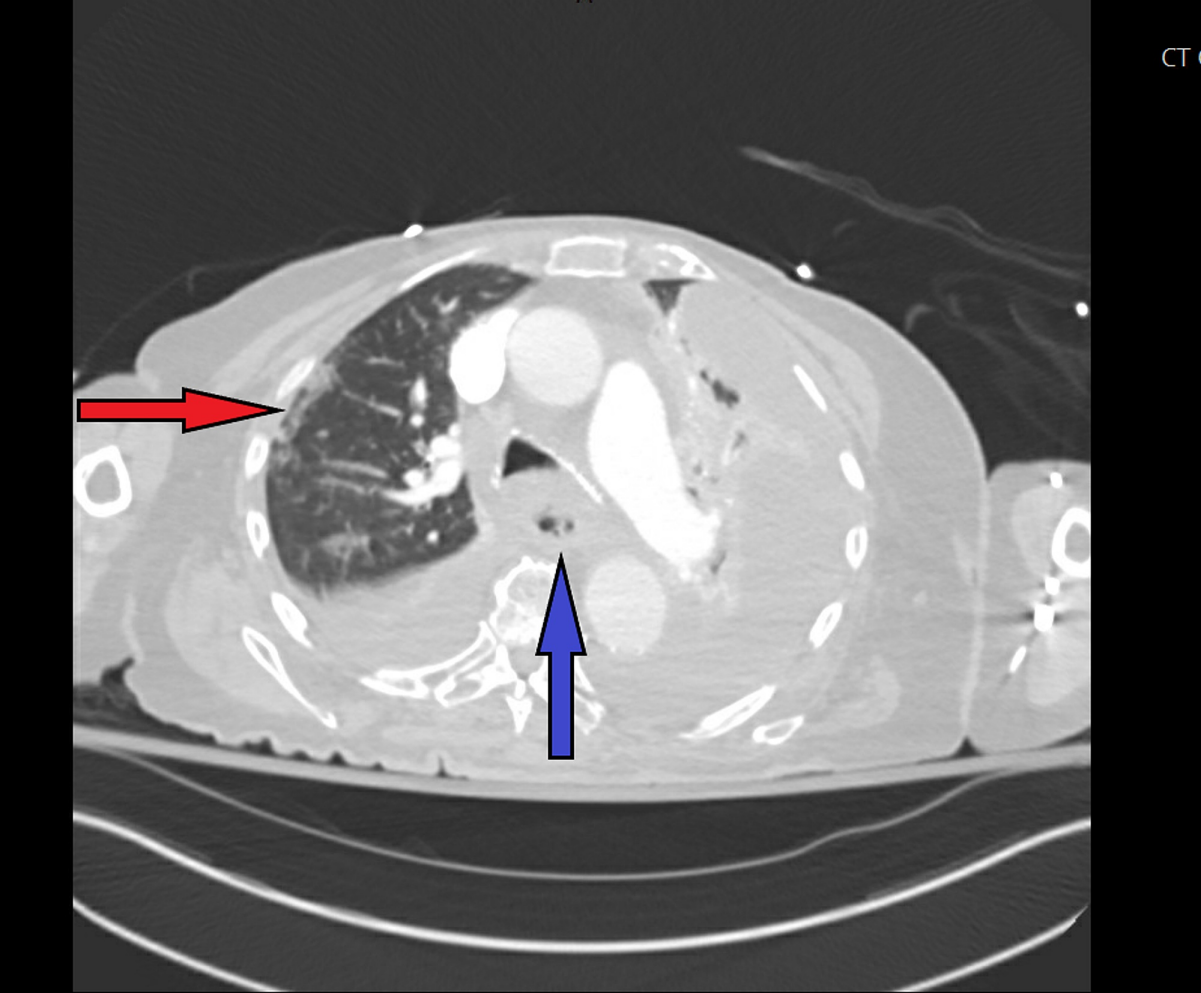
CT angiogram of the chest with multiple pathologic findings Selected image from the patient's CT angiogram of the chest that reveals ground-glass opacities (red arrow) of the right lung field, which is consistent with viral/COVID-19 pneumonia, and an extraluminal air collection (blue arrow) next to the esophagus indicating acute esophageal rupture CT: computed tomography; COVID-19: coronavirus disease 2019

After seven hours in the ED, the patient was transferred to the medical floor, and had a large maroon bowel movement with blood clots, and was transferred to the ICU for resuscitation. The patient was found to be COVID-19-positive, and an urgent endoscopy was completed, which revealed an acute Mallory-Weiss tear with fresh bleeding. There was no hiatal hernia, or any other pathology noted on endoscopy. The patient was stabilized initially, and a repeat CT chest, abdomen, and pelvis revealed diffuse ground-glass opacities in the lung fields consistent with “viral pneumonia”, as well as an acute esophageal rupture (Figure 1).

Subsequently, after an extensive conversation about the patient's prognosis with her family, her code status was changed to do-not-resuscitate/do-not-intubate (DNR/DNI), and she died due to the above-mentioned complications.

## Discussion

The COVID-19 pandemic has been recognized as both a short- and long-term health crisis, which has stretched health resources to the limit globally. Though initial reports of infectious symptoms seemed to be limited to fever, cough, and shortness of breath, with an associated bilateral viral pneumonia [4], additional studies have revealed that 20% of patients also had one gastrointestinal symptom, which includes diarrhea, vomiting, or abdominal pain [6]. Ongoing research on the evolving nature of the pandemic has also revealed concerning findings of increased venous thromboembolism (VTE) as well as increased bleeding events [7] in hospitalized patients. Though routine hospital admissions typically involve anticoagulation to prevent VTE, standard therapies have not been effective in preventing these VTE events, and ongoing trials are exploring alternate anticoagulation dosing and therapies.

Boerhaave's syndrome is clinically characterized by sudden esophageal perforation, most commonly caused by an iatrogenic injury or forceful vomiting, and has a high mortality rate, ranging from 20-50% [8,9]. This case is unique for its association with violent coughing fits, which has been rarely reported in the Boerhaave syndrome literature. The incidence of Boerhaave syndrome is remarkably low, 3.1 per 1,000,000 persons, and clinically, most patients who develop Boerhaave's syndrome present with acute dyspnea and chest pain, and it should be suspected in patients in whom overeating and violent vomiting are noted [9]. Signs of sepsis or shock, including fever, tachycardia, and hypotension, are often seen late in the disease process [10]. Differential diagnoses of this type of presentation also include acute coronary syndrome, pneumonia, or pulmonary embolism, and this broad differential scope can lead to a delay in diagnosis, which likely contributes to its high mortality rate [11,12].

In this case, we believe our patient's age and comorbid condition of COVID-19 contributed directly to the initial development of Boerhaave syndrome and her rapid decompensation. Though she was appropriately resuscitated initially and underwent extensive evaluation for her pain, she continued to experience symptoms of COVID-19, including increasing coughing and vomiting. These ongoing symptoms of coughing and vomiting were the primary contributors to the esophageal tear and rupture, which was the direct cause of her death.

## Conclusions

Despite early and intensive management of our patient’s abdominal pain and hypotension, she developed a Mallory-Weiss tear and Boerhaave syndrome after hospitalization. COVID-19 was found to be the primary driver of her violent coughing spells, vomiting, and was likely the culprit behind her increased susceptibility to bleeding while hospitalized. This case illustrates the ongoing challenge that healthcare providers need to address during the COVID-19 pandemic. Though clinicians need not suspect Mallory-Weiss tear or Boerhaave syndrome for each presentation of COVID-19, an awareness of the various complications from COVID-19 is crucial to making an urgent diagnosis and treatment plan for all hospitalized patients.
